# Columbianadin ameliorates experimental acute reflux esophagitis in rats via suppression of NF-κB pathway

**DOI:** 10.1590/acb391824

**Published:** 2024-05-03

**Authors:** Ying Wu, Shaik Althaf Hussain, Minghai Luo

**Affiliations:** 1 The Second People’s Hospital of Shaanxi Province – Department of Gastroenterology – Xi ‘an, China.; 2 King Saud University – College of Science – Department of Zoology – Riyadh, Saudi Arabia.; 3 Ankang City Central Hospital – Department of Pediatric – AnKang, China.

**Keywords:** Esophagitis, Peptic, Oxidative Stress, Inflammation, Cell Survival

## Abstract

**Purpose:**

Reflux esophagitis is a condition characterized by inflammation and irritation of the esophagus, resulting from the backflow of stomach acid and other gastric contents into the esophagus. Columbianadin is a coumarin derivative that exhibits anti-inflammatory and antioxidant effects. In this study, we tried to scrutinize the protective effect of Columbianadin against acute reflux esophagitis in rats.

**Methods:**

RAW 264.7 cells were utilized to assess cell viability and measure the production of inflammatory parameters. The rats received anesthesia, and reflux esophagitis was induced via ligation of pylorus and fore stomach and corpus junction. Rats received the oral administration of Columbianadin (25, 50 and 100 mg/kg) and omeprazole (20 mg/kg). The gastric secretion volume, acidity, and pH were measured. Additionally, the levels of oxidative stress parameters, cytokines, and inflammatory markers were determined. At the end of the study, mRNA expression was assessed.

**Results:**

Columbianadin remarkably suppressed the cell viability and production of tumor necrosis factor-α (TNF-α), interleukin (IL)-1β, IL-6, cyclooxygenase-2 (COX-2), inducible nitric oxide synthase (iNOS), and prostaglandin (PGE2). Columbianadin treatment remarkably suppressed the secretion of gastric volume, total acidity and enhanced the pH level in the stomach. Columbianadin remarkably altered the level of hydrogen peroxidase, free iron, calcium, and plasma scavenging activity, sulfhydryl group; oxidative stress parameters like malonaldehyde, glutathione, superoxide dismutase, catalase, glutathione peroxidase; inflammatory cytokines viz., TNF-α, IL-6, IL-1β, IL-10, IL-17, and monocyte chemoattractant protein-1; inflammatory parameters including PGE_2_, iNOS, COX-2, and nuclear kappa B factor (NF-κB). Columbianadin remarkably (P < 0.001) suppressed the mRNA expression TNF-α, IL-6, IL-1β and plasminogen activator inhibitor-1.

**Conclusions:**

Columbianadin demonstrated a protective effect against acute reflux esophagitis via NF-κB pathway.

## Introduction

Reflux esophagitis (RE) is a significant manifestation of gastroesophageal reflux disease (GERD) that can have a substantial impact on a patient’s quality of life^1^. It may manifest with symptoms such as heartburn (a burning sensation in the chest), regurgitation of stomach contents into the mouth, difficulty swallowing, and chest pain. These symptoms can significantly impact daily life. Without proper treatment or management, RE can progress to more serious complications^2^^,^ 3.

Chronic inflammation and injury to the lining of the esophagus can lead to several complications, including esophageal narrowing (stricture), Barrett’s esophagus (a condition associated with a higher risk of esophageal cancer), and erosive esophagitis. GERD is a chronic condition characterized by the backflow of stomach acid and sometimes stomach contents into the esophagus, resulting in irritation and inflammation. RE occurs when this reflux causes inflammation and damage to the esophageal lining^2^^,^ 3. The symptoms and potential complications of RE can significantly affect a patient’s quality of life. Chronic heartburn and discomfort can make it challenging to enjoy meals, sleep comfortably, and participate in social activities.

The disease occurs due to poor habit, stress, food and smoking^4^. Prolonged and severe GERD can cause various types of esophageal mucosal injury, including bleeding, erythema (redness), erosion, and ulcers. These injuries can be painful and may lead to complications if left untreated^2^.

There are several types of medications used to manage GERD and alleviate its symptoms. Antacids are over-the-counter medications that work by neutralizing stomach acid, providing quick relief from heartburn and acidity4. Acid blockers are drugs that reduce the production of stomach acid. Histamine type 2 (H_2_) antagonists such as ranitidine and cimetidine work by blocking the action of histamine on stomach cells, which in turn reduces acid production. Proton pump inhibitors like omeprazole and esomeprazole are among the most widely used therapies for GERD. They work by inhibiting the proton pump (proton-transporting enzyme) in the stomach lining, which drastically reduces acid secretion. These medications can help improve the movement of the stomach and reduce the risk of acid reflux into the esophagus^2^^,^ 5.

In some severe cases of GERD that do not respond well to medication, surgical procedures may be considered to address the issue, such as fundoplication. H_2_ antagonists and proton pump inhibitors are two classes of acid-blocking medications commonly used in the management of GERD. H_2_ antagonists, these drugs, like ranitidine and cimetidine, reduce stomach acid production by blocking histamine signals in the stomach^2^. These medications are typically prescribed based on the severity of GERD symptoms and the patient’s response to treatment. Lifestyle modifications, such as dietary changes and elevating the head of the bed, are also important aspects of managing GERD in addition to medication. Patients with GERD should consult with a healthcare professional for proper diagnosis and treatment recommendations^2^^,^ 4.

 The underlying mechanism of RE is not fully understood, but few reports suggest that lipid peroxidation and free radical production play a crucial role in the gastroesophageal disease. Few studies suggest that the increased level of reactive oxygen species (ROS) is directly linked with the esophageal lesions, leading to the production of lipid peroxidation (LPO) in the membranes via oxidative of unsaturated fatty acid^6^^,^ 7. This study on rats found that oxygen-based molecules that damage cells cause esophageal injury and increase the breakdown of fats in the esophageal lining. It also found that giving different substances that remove these molecules can prevent esophageal injury caused by the backflow of stomach and intestinal fluids, but lowering the acid level with ranitidine alone cannot reduce the severity of RE or the inflammation linked to the activation of a protein called nuclear factor-κ B (NF-κB) by these molecules^5^^,^ 8.

Ranitidine is a medication known as an H2 blocker that reduces stomach acid production. While it can be effective in alleviating symptoms of acid reflux, it may not be as effective in preventing or treating RE caused by mixed reflux with non-acidic contents^5^^–^ 7. Oxygen-based molecules that damage cells are very important in causing diseases in different tissues like the digestive system7. Free radicals act as a carcinogens, since they lead to DNA injury. Also, free radicals induce the esophageal mucosa or gastric injury^9^.

It has been showed that oxygen derived free radicals induces the esophageal mucosal and acute gastric injury due to ischemia, ethanol or non-steroidal anti-inflammatory drugs (NSAIDs)^5^. The mostly available treatment for the RE is NSAIDS. The NSAIDS are commonly used as antipyretic, anti-inflammatory, analgesic and anti-rheumatic and mostly in the treatment of fever, pain, and arthritis^10^^,^ 11. Therefore, NSAIDS have excellent anti-inflammatory effects, but these drugs have serious side effects such as causes of liver injury, gastrointestinal dyspepsia, allergies, and other ones^4^.

Columbianadin is a natural compound found in certain plants, particularly in the genus *Angelica*, which includes *Angelica keiskei Koidzumii* and *Angelica gigas* Nakai. It is classified as a coumarin derivative, a type of organic compound commonly found in plants^12^^,^ 13. Columbianadin has been subject of interest in research due to its potential pharmacological properties. Some studies have suggested that it may have anti-inflammatory, antioxidant, and anti-cancer properties^12^^–^ 15. However, further research is needed to fully understand its mechanisms of action and its potential applications in medicine.

In this study, we tried to explore the protective effect of acute RE in rats and the underlying mechanism.

## Methods

### >Animals and treatment

#### *In vitro*: cell culture and viability

For the *in-vitro* study, we used the ATCC RAW 264.7 macrophage cells. The cells were propagated in the Dulbecco’s modified eagle medium (DMEM) with 10% fetal bovine serum (FBS) and 1% P/S in an incubator at 37°C. The cells were cultured in the medium for seven days, and all alternate day the medium was replaced. After the seven days, the cells were supplemented with Columbianadin and incubated with 1-µg/mL lipopolysaccharide (LPS) for 24 h.

We followed the manufacturer’s instructions (Dojindo Molecular Technology, Inc., Rockville, MD, United States of America) to use a cell counting kit (CCK-8) to measure how Columbianadin affects the cells. We put the cells in a 96-well plate and exposed them to different amounts of Columbianadin (5, 10, 20 and 40 µM) for 24 h. Then, we used a microplate reader to check how much light they absorbed at 450 nm.

#### Nitric oxide production

For the determination of nitric oxide (NO) production, the RAW 264.7 cells (2.5 × 10^4^ cells/mL) were incubated in 96-well plate and treated with LPS (1 µg/mL) alone or with Columbianadin for 24 h. After that, the culture supernatants were successfully removed, and the equal concentration of Griess reagent was added and further incubated for 10 min at 37°C. Finally, estimation the absorbance at 540 nm using the microplate reader was performed.

#### Cytokines and inflammatory parameters

We put the cells in the same way as before and measured how much of the inflammation-related substances like tumor necrosis factor-α (TNF-α), interleukin (IL)-1β, IL-6, cyclooxygenase-2 (COX-2), prostaglandin (PGE2), inducible nitric oxide synthase (iNOS), and NF-κB they had. We used a kit that tests how well the substances compete with enzymes and followed the maker’s directions (R&D Systems Inc., Minneapolis, MN, United States of America).

### Experimental rodent

Swiss albino Wistar rats sex either male, aged 10–12 weeks old, weight 150 ± 25 g, were used in the protocol. The whole procedure was carried out accordance to ethics committee of the university and animal care via following the recommendation of the committee. The rats were provided with a standard pellet diet and water *ad libitum*. They were housed under standard laboratory conditions, in a temperature of 20 ± 5°C, relative humidity of 60%, and a 12/12-h light/dark cycle. The rats were acclimatized to the laboratory environment for seven days prior to the commencement of the experiment.

#### Experimental design

After the acclimatization of rats, they were divided into 6 groups, each group contains six rats. The groups were divided as follow:

- Group A: normal control (orally received the physiological solution);
- Group B: RE control;
- Group C: RE + Columbianadin (25 mg/kg);
- Group D: RE + Columbianadin (50 mg/kg);
- Group E: RE + Columbianadin (100 mg/kg);
- Group F: RE + omeprazole (20 mg/kg).

Group B to Group F rats had the RE induced. After 1 h, the rats received anesthesia using the phenobarbitone, and celotomy was carried out to induce the RE via ligation of pylorus and fore stomach and corpus junction using silk sutures (2-0), in accordance with the previous published literature. During the induction of RE, the rats were free from food and water.

#### Sample collection

The rats were anesthetized, and blood was collected by puncturing the retro-orbital plexus. The collected blood samples were then centrifuged at 10,000 rpm for 10 minutes at 4°C to separate the serum, which was subsequently stored at -20°C for determination of biochemical parameters.

After 6 h, the double ligation was carried out for the autopsy of experimental rats. The small part of gastroesophageal digestive tract was quickly removed and inspected to scrutinize its appearance, and phosphate buffered saline (PBS) was used for homogenization. After that, the supernatant was collected to scrutinize the different biochemical parameters.

#### Gastric acid analysis

For the estimation of gastric content, the gastric contents were collected in conical tubes (15 mL) and centrifuged for 10 min at 3,000 × g. After the centrifugation, the supernatant was collected in the conical tubes (1.5 mL), and acidity (μEq/L) and its volume (mL) were estimated. The acidity was determined using pH meter by titration with NaOH (0.1 N) at pH 7.

#### Cytokines and inflammatory parameters

The level of inflammatory cytokines, TNF-α, IL-1β, IL-6, IL-10, IL-17, and chemokine (MCP-1) were estimated with multi-analyte enzyme-linked immunosorbent assay (ELISA) kit following the manufacture instruction (Millipore, Rockford, IL, United States of America).

The inflammatory parameters like COX-2, PGE2, iNOS and NF-κB were estimated using the multi-analyte ELISA kit following the manufacture instruction (Millipore, Rockford, IL, United States of America).

#### mRNA expression

Total RNA was isolated from the intestinal graft obtained from the esophagus using TRIzol reagent (Invitrogen Life Technologies, Inc., Grand Island, NY, United States of America) according to the manufacturer’s instructions. The concentration of RNA was determined using ultraviolet (UV) spectrophotometry. For quantitative polymerase chain reaction (qPCR) analysis, the SYBR Green PCR kit was utilized following a previously reported method. Thermal cycling conditions involved 15 seconds at 95°C and 1 minute at 60°C using the ABI PRISM 7000 Sequence Detection System (Applied Biosystems). The primer sequences are listed in Table 1, and glyceraldehyde 3-phosphate dehydrogenase (GAPDH) was used as the internal standard.

**Table 1:** List of primer.

S. No	Primer	Sequence (5'- 3')	
		Reverse	Forward
1	TNF-α	TCATACCAGGGCTTGAGCTCA	CCAGGAGAAAGTCAGCCTCCT
2	IL-1β	GGGTTCCATGGTGAAGTCAAC	CACCTCTCAAGCAGAGCACAG
3	IL-6	GGCAACTGGCTGGAAGTCTCT	CGAAAGTCAACTCCATCTGCC
4	PAI-1	TTGTCTGATGAGTTCAGCATCCA	CCGATGGGCTCGAGTATGA
5	GAPDH	CGCTCCTGGAAGATGGTGAT	ATGGCACAGTCAAGGCTGAGA

TNF-α: tumor necrosis factor-α; IL: interleukin; PAI-1: plasminogen activator inhibitor-1; GAPDH: glyceraldehyde 3-phosphate dehydrogenase.

### Statistical analyses

Statistical analysis was conducted using GraphPad Prism software 8 (St. Louis, United States of America). Results are expressed as the mean ± standard error of the mean (SEM). One-way analysis of variance (ANOVA) followed by Dunnett’s t-test was employed, with P < 0.05 considered statistically significant.

## Results

### Cell viability

**Figure 1:**
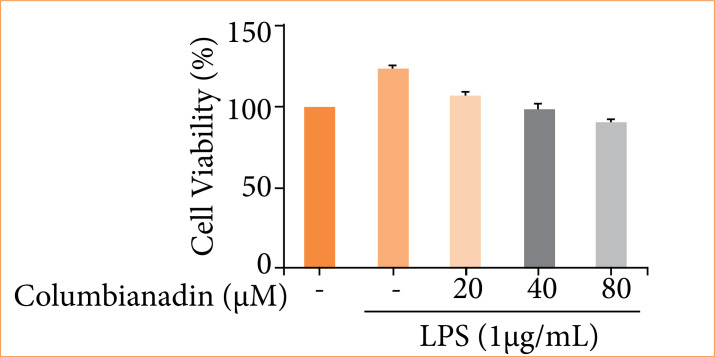
Effect of Columbianadin on the cell viability in RAW 264.7 cells*.

For scrutinize the effect of Columbianadin on the cell growth, the RAW 264.7 cells were treated with the different concentration of Columbianadin (0, 20, 40 and 80 μM) up to 24 h. Figure 1 exhibits the effect of Columbianadin on the cell viability. Columbianadin did not exhibit any effect on the growth of normal cells. The various concentration of Columbianadin showed the effect on the RAW 267.4 cells.

LPS: lipopolysaccharide; *data are presented as mean ± standard deviation; ###p < 0.001 compared with normal control cells. Source: Elaborated by the authors.

### Inflammatory cytokines and parameters

**Figure 2:**
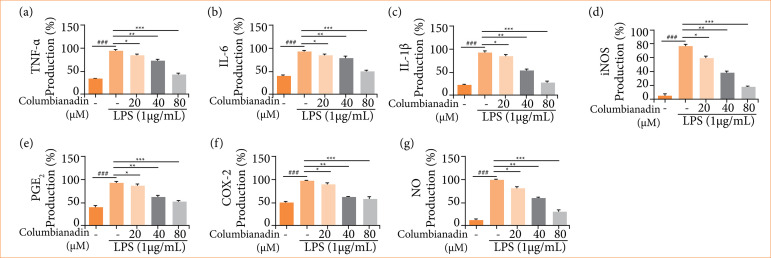
Effect of Columbianadin on the inflammatory cytokines and inflammatory parameters on LPS induced RAW 264.7 cells. **(a)** TNF-α, **(b)** IL-6, **(c)** IL-1β, **(d)** iNOS, **(e)** PGE_2_, **(f)** COX-2, **(g)** NO.

Figure 2 exhibits the reduction in the production of TNF-α (Fig. 2a), IL-6 (Fig. 2b), IL-1β (Fig. 2c), iNOS (Fig. 2d), PGE2 (Fig. 2e), COX-2 (Fig. 2f), and NO (Fig. 2g) against the LPS treated by Columbianadin. The level of inflammatory cytokines and parameters were suppressed by the Columbianadin at a dose-dependent manner.

TNF-α: tumor necrosis factor-α; LPS: lipopolysaccharide; IL: interleukin; iNOS: inducible nitric oxide synthase; PGE_2_: prostaglandin; COX-2: cyclooxygenase-2; NO: nitric oxide; !data are presented as mean ± standard deviation; ###p < 0.001 compared with normal control cells; *p < 0.05, **p < 0.01 and ***p < 0.001 compared with normal control cells. Source: Elaborated by the authors.

### Gastric secretion, total acidity, and pH

**Figure 3:**
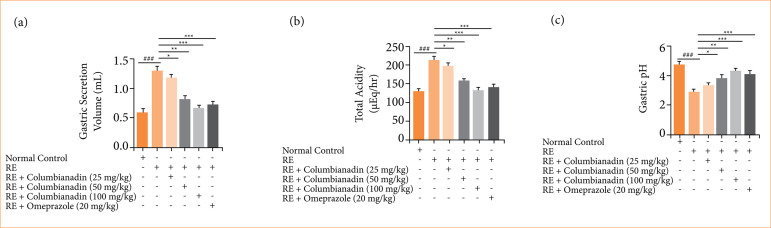
Effect of Columbianadin on the gastric secretion volume, total acidity, and pH against acute reflux esophagitis in rats!. **(a)** Gastric secretion volume, **(b)** total acidity, **(c)** pH.

RE induced group rats demonstrated the boosted gastric secretion volume (Fig. 3a), total acidity (Fig. 3b), and decreased pH (Fig. 3c) level. Columbianadin and omeprazole remarkably decreased the level of gastric secretion volume, total acidity, and boosted the pH.

RE: reflux esophagitis; !data are presented as mean ± standard deviation; ###p < 0.001 compared with normal control cells; *p < 0.05, **p < 0.01 and ***p < 0.001 compared with normal control cells. Source: Elaborated by the authors.

### H_2_O_2_, free iron, calcium, plasma scavenging activity and sulfhydryl group

**Figure 4:**
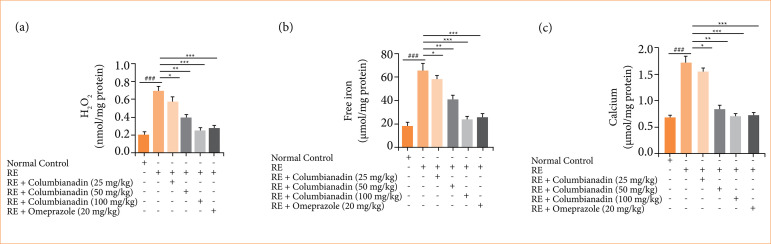
Effect of Columbianadin on the H_2_O_2_, free iron, and calcium against acute reflux esophagitis in rats!. **(a)** Gastric secretion volume, **(b)** total acidity, **(c)** calcium.

The levels of H_2_O_2_ (Fig. 4a), free iron (Fig. 4b), and calcium (Fig. 4c) in the RE group rats and Columbianadin and omeprazole treatment were significantly (P < 0.001) suppressed.

RE: reflux esophagitis; !data are presented as mean ± standard deviation; ###p < 0.001 compared with normal control cells; *p < 0.05, **p < 0.01 and ***p < 0.001 compared with normal control cells. Source: elaborated by the authors

**Figure 5:**
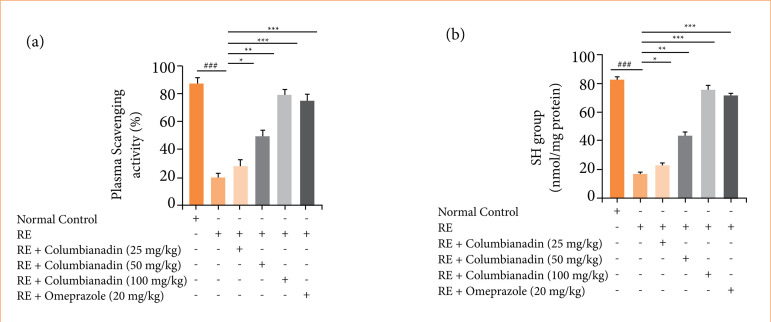
Effect of Columbianadin on the plasma scavenging activity and SH group against acute reflux esophagitis in rats. **(a)** Plasma scavenging activity, **(b)** SH group.

RE: reflux esophagitis; SH: sulfhydryl; !data are presented as mean ± standard deviation; ###p < 0.001 compared with normal control cells; *p < 0.05, **p < 0.01 and ***p < 0.001 compared with normal control cells. Source: Elaborated by the authors.

RE group rats exhibited the suppressed level of plasma scavenging activity (Fig. 5a), sulfhydryl (SH) group (Fig. 5b), and Columbianadin and omeprazole treatment remarkably (P < 0.001) boosted the level.

### Antioxidant parameters

**Figure 6:**
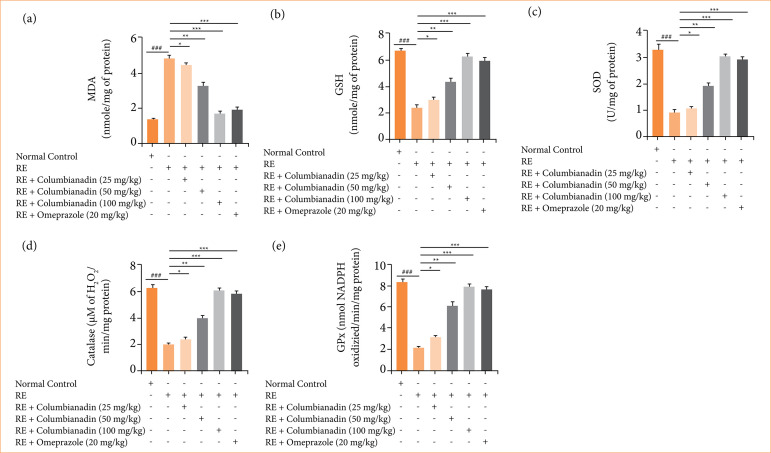
Effect of Columbianadin on the antioxidant parameters against acute reflux esophagitis in rats. **(a)** MDA, **(b)** GSH, **(c)** SOD, **(d)** catalase, **(e)** GPx.

RE: reflux esophagitis; MDA: malonaldehyde; GSH: glutathione; SOD: superoxide dismutase; !data are presented as mean ± standard deviation; ###p < 0.001 compared with normal control cells; *p < 0.05, **p < 0.01 and ***p < 0.001 compared with normal control cells. Source: Elaborated by the authors.

RE group rats revealed the boosted level of malonaldehyde (MDA) (Fig. 6a) and suppressed the levels of glutathione (GSH) (Fig. 6b), superoxide dismutase (SOD) (Fig. 6c), catalase (CAT) (Fig. 6d), and glutathione peroxidase (GPx) (Fig. 6e), and Columbianadin and omeprazole treatment remarkably (P < 0.001) restored the level of antioxidant parameters.

### Inflammatory cytokines and inflammatory parameters

**Figure 7:**
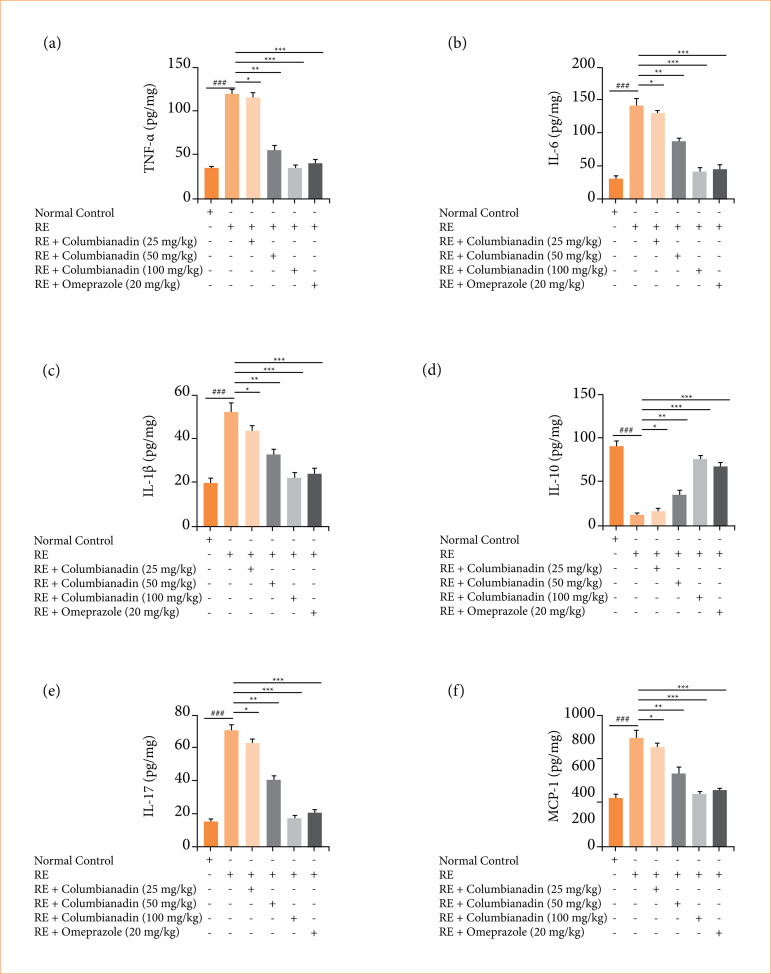
Effect of Columbianadin on the cytokines against acute reflux esophagitis in rats. **(a)** TNF-α, **(b)** IL-6, **(c)** IL-1β, **(d)** IL-10, **(e)** IL-17, **(f)** MCP-1.

RE: reflux esophagitis; TNF-α: tumor necrosis factor-α; IL: interleukin; MCP-1: monocyte chemoattractant protein-1; !data are presented as mean ± standard deviation; ###p < 0.001 compared with normal control cells; *p < 0.05, **p < 0.01 and ***p < 0.001 compared with normal control cells. Source: Elaborated by the authors.

RE group rats revealed the altered level of TNF-α (Fig. 7a), IL-6 (Fig. 7b), IL-1β (Fig. 7c), IL-10 (Fig. 7d), IL-17 (Fig. 7e), and MCP-1 (Fig. 7f), and Columbianadin remarkably (P < 0.001) modulated the level of inflammatory cytokines. 

**Figure 8:**
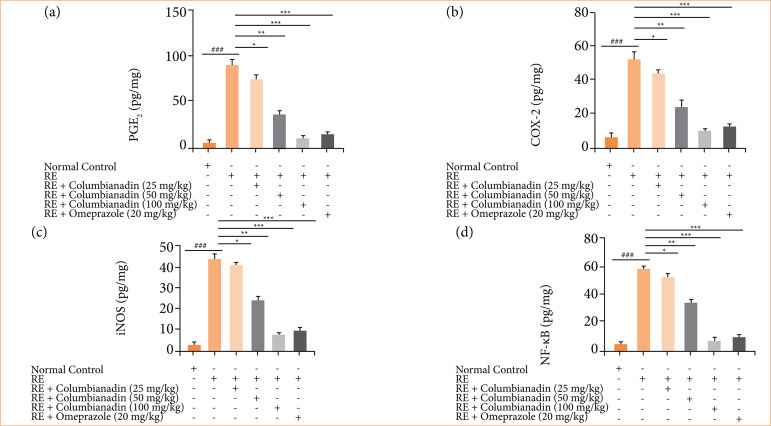
Effect of Columbianadin on the inflammatory parameters against acute reflux esophagitis in rats. **(a)** PGE2, **(b)** COX-2, **(c)** iNOS, **(d)** NF-κB.

RE: reflux esophagitis; PGE_2_: prostaglandin; COX-2: cyclooxygenase-2; iNOS: inducible nitric oxide synthase; NF-κB: nuclear kappa B factor; !data are presented as mean ± standard deviation; ###p < 0.001 compared with normal control cells; *p < 0.05, **p < 0.01 and ***p < 0.001 compared with normal control cells. Source: Elaborated by the authors.

RE group rats demonstrated the boosted level of PGE2 (Fig. 8a), COX-2 (Fig. 8b), iNOS (Fig. 8c), and NF-κB (Fig. 8d), and Columbianadin remarkably (P < 0.001) down-regulated the level of inflammatory parameters.

### mRNA expression

**Figure 9:**
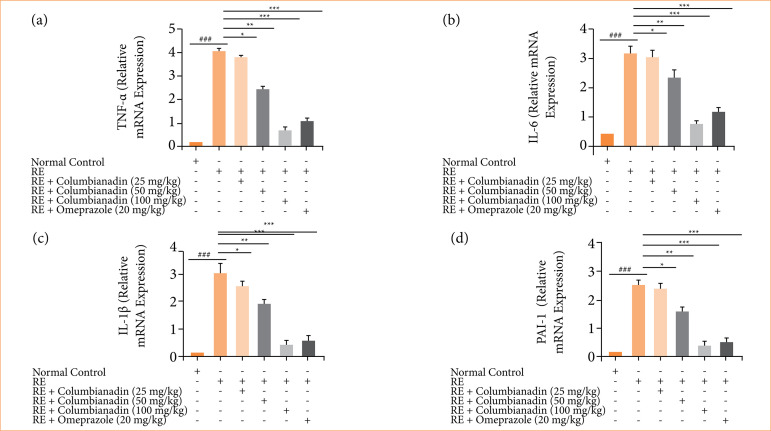
Effect of Columbianadin on the mRNA expression against acute reflux esophagitis in rats. **(a)** TNF-α, **(b)** IL-6, **(c)** IL-1β, **(d)** PAI-1.

RE: reflux esophagitis; TNF-α: tumor necrosis factor-α; IL: interleukin; PAI-1: plasminogen activator inhibitor-1; !data are presented as mean ± standard deviation; ###p < 0.001 compared with normal control cells; *p < 0.05, **p < 0.01 and ***p < 0.001 compared with normal control cells. Source: Elaborated by the authors.

RE group rats demonstrated the boosted mRNA expression of TNF-α (Fig. 9a), IL-6 (Fig. 9b), IL-1β (Fig. 9c), and PAI (Fig. 9d), and Columbianadin and omeprazole treatment remarkably (P < 0.001) restored the mRNA expression.

## Discussion

RE is a condition that often requires long-term medication, and it can have a high recurrence rate. It is characterized by inflammation and damage to the esophagus caused by repeated episodes of gastroesophageal reflux, in which stomach acid flows back into the esophagus^4^. The symptoms of RE can be uncomfortable and may include heartburn, chest pain, difficulty swallowing, and regurgitation. Phytomedicine, which includes herbal remedies and natural plant-based medicines, has gained attention as an alternative or complementary approach to managing RE. Some herbs and plants are believed to have anti-inflammatory and soothing properties that may help alleviate symptoms. Traditional herbal medicine systems, such as traditional Chinese medicine, have used various herbal remedies for digestive disorders, including RE. These remedies are often based on centuries-old practices and are believed to help balance the body and alleviate symptoms.

RE is characterized by the inflammation and irritation of the esophageal lining due to the backflow of stomach contents, including stomach acid. Inflammation is a complex process involving various molecular and cellular components, and several inflammatory mediators, including TNF-α, IL-1β, IL-6, iNOS, COX-2, PGE2, and NO can play roles in the pathogenesis of RE^4^^,^ 16. The collective actions of these inflammatory mediators contribute to the inflammation, tissue damage, and symptoms associated with RE.

TNF-α is a proinflammatory cytokine produced by immune cells. In RE, TNF-α can be released due to tissue damage and inflammation in the esophagus. It promotes inflammation by activating immune cells and promoting the production of other inflammatory mediators^4^^,^ 17. IL-1β, a proinflammatory cytokine, is synthesized by various cell types, including immune cells and epithelial cells. Its presence facilitates the recruitment of immune cells and initiates the inflammatory response within the esophagus. IL-6 is a cytokine with diverse functions, playing a crucial role in both inflammation and immune responses. It can be produced in response to tissue damage and inflammation caused by reflux of stomach contents into the esophagus. IL-6 can amplify the inflammatory response in the esophagus. iNOS is an enzyme that produces NO, a molecule involved in various physiological processes. In RE, iNOS can be upregulated in response to inflammation^2^^,^ 4 ^,^ 17 ^,^ 18. NO produced by iNOS can contribute to tissue damage and inflammation. COX-2 is an enzyme involved in the production of prostaglandins, including PGE_2_. PGE2 has proinflammatory effects. In RE, COX-2 and PGE_2_ can be elevated in response to inflammation and tissue damage, contributing to the inflammatory process^2^. NO is a signaling molecule that can have both proinflammatory and anti-inflammatory effects depending on its concentration and context. In RE, NO produced by iNOS can promote inflammation and tissue damage 4 ^,^ 18.

RE is a condition characterized by the backflow of stomach acid and other gastric contents into the esophagus, causing irritation and inflammation of the esophageal lining. Several factors related to gastric secretion can contribute to the development and severity of RE. Gastric secretion volume refers to the amount of gastric acid and other fluids produced by the stomach^4^. An increased volume of gastric secretion can contribute to RE by providing more acid to flow back into the esophagus. Larger volumes of gastric secretion increase the likelihood of more acid reaching the lower esophagus, potentially causing more severe irritation and damage. Gastric pH measures the acidity of the stomach^5^. A lower pH value indicates a more acidic environment. When the pH of gastric contents is lower (more acidic), it is more likely to cause damage to the esophagus when reflux occurs. Lowering gastric pH increases the corrosive potential of refluxed stomach acid, making it more harmful to the esophageal lining. Total acidity refers to the overall acidic content in the stomach, which includes not only hydrochloric acid, but also other components like pepsin. High levels of total acidity can contribute to the severity of RE, because it amplifies the corrosive effects of stomach contents on the esophagus^2^^,^ 5.

RE is primarily characterized by the irritation and inflammation of the esophageal lining due to the backflow of stomach contents, including stomach acid. While plasma scavenging activity, SH groups (sulfhydryl groups), H_2_O_2_ (hydrogen peroxide), free iron, and calcium are important factors in various physiological processes; their direct roles in RE are not as well-established as other factors like gastric acid and inflammation^2^^,^ 19. Plasma scavenging activity typically refers to the ability of antioxidants and other compounds to neutralize harmful free radicals and ROS in the bloodstream. In the context of RE, oxidative stress resulting from the reflux of gastric contents can contribute to tissue damage in the esophagus. Antioxidants in the bloodstream, including those from the diet, may help mitigate some of the oxidative damage caused by refluxed substances^19^.

SH groups are functional groups containing sulfur and hydrogen atoms. They are present in various proteins and enzymes in the body and play roles in maintaining protein structure and function. While SH groups are important in cellular processes, their direct involvement in RE is not a well-known aspect of the condition. H_2_O_2_ is a reactive oxygen species that can be produced as part of the oxidative stress response. It can contribute to tissue damage and inflammation when present in excess^2^. It is possible that H_2_O_2_, along with other ROS, could play a role in the inflammation and damage seen in RE, but further research is needed to establish this connection. Free iron and calcium are essential minerals in the body with diverse roles in various physiological processes. Iron, particularly, can act as a pro-oxidant when present in excess and may contribute to oxidative stress. Calcium, on the other hand, plays a role in muscle contraction, including the function of the lower esophageal sphincter. Alterations in calcium and iron levels in the context of RE may indirectly affect symptoms and contribute to esophageal dysfunction^2^^,^ 4.

RE is primarily characterized by the irritation and inflammation of the esophageal lining due to the backflow of stomach contents, including stomach acid. While MDA, SOD, GSH, GPx, and CAT are important factors in various physiological processes, their direct roles in RE are not as well-established as other factors like gastric acid and inflammation^20^. MDA is a marker of lipid peroxidation, a process that occurs when oxidative stress damages cell membranes and lipids. Oxidative stress resulting from refluxed gastric contents may contribute to tissue damage in the esophagus. MDA levels can increase because of oxidative stress, but its specific role in RE is not extensively studied. SOD is an antioxidant enzyme that plays a critical role in scavenging and neutralizing superoxide radicals and other ROS^2^^,^ 21. In RE, oxidative stress can lead to the generation of ROS, and SOD may help counteract some of this oxidative damage. GSH is a vital antioxidant found in cells that helps protect against oxidative stress and detoxify harmful compounds. GSH can be depleted in response to oxidative stress. While GSH plays a significant role in cellular protection, its direct involvement in RE is not extensively studied^20^^,^ 22 ^–^ 24. GPx is an enzyme that works in concert with GSH to neutralize peroxides and protect cells from oxidative damage. In the context of RE, it may help mitigate some of the oxidative stress caused by refluxed substances, but its specific role is not well-documented. CAT is another enzyme involved in the breakdown of hydrogen peroxide into water and oxygen. Similar to SOD and GPx, it can help reduce oxidative stress in cells^1^^,^ 25 ^,^ 26.

## Conclusion

Columbianadin suppressed the cell viability against the RAW 264.7 cells along with reduction of the production of TNF-α, IL-6, IL-1β, iNOS, PGE2, and COX-2. Columbianadin remarkably suppressed the gastric secretion volume, total acidity and boosted the pH level. Columbianadin remarkably altered the level of antioxidant, cytokines, and inflammatory parameters along with restore the mRNA expression.

In future, we estimate the molecular mechanism for the estimation of protective effect of Columbianadin against RE.

## Data Availability

The data will be available upon request.
